# Supplementary material for: Enhancing the efficiency of time-dependent density functional theory calculations of dynamic response properties
Source: NPJ Comput Mater. 2026 Apr 25;12(1):168. doi: 10.1038/s41524-026-02088-9 (PMC13110131; doi:10.1038/s41524-026-02088-9)
Supplement: Supplementary file 1 — Supplementary Information [file 41524_2026_2088_MOESM1_ESM.pdf]

Supplemental Material: Enhancing the Efficiency of Time-Dependent Density Functional Theory Calculations of Dynamic Response Properties

Zhandos A. Moldabekov[1*], Sebastian Schwalbe[1], Uwe Hernandez Acosta[1], Thomas Gawne[1,2], Jan Vorberger[1], Michele Pavanello[3] and Tobias Dornheim[1,2]

[1] Institute of Radiation Physics, Helmholtz-Zentrum Dresden-Rossendorf (HZDR), Dresden, D-01328, Germany.
[2]Center for Advanced Systems Understanding (CASUS), Görlitz, D-02826, Germany.
[3]Department of Physics, Rutgers University, Newark, NJ 07102, USA.

*Corresponding author. E-mail: z.moldabekov@hzdr.de;

The Kubo-Greenwood method is commonly employed to characterize the dynamic transport properties of materials under extreme conditions in the optical limit [1–5]. Given the importance of this approach for such applications as warm dense matter and dense plasmas, we outline the steps to implement the η-convergence test in the imaginary time domain alongside a constraints-based filtering starting from the Kubo-Greenwood conductivity $\sigma_{\mathrm{KG}}(\omega)$. In this context, we consider the averaged value of $\sigma_{\mathrm{KG}}(\omega)$ defined as the trace of the conductivity tensor [4].

Using Eq. (10) and Eq. (11) from the main paper, one can express the imaginary part of the dynamic density response function in terms of the dynamic conductivity $\sigma_{\mathrm{KG}}(\omega)$ as

$$\chi^{*}_{\mathrm{KG}}(\omega) \underset{\vec{q}\to 0}{=\!=} v(q)\chi_{\mathrm{KG}}(\vec{q},\omega) \equiv \mathrm{Im}\left[\frac{1}{1+\frac{4\pi i}{\omega}\sigma_{\mathrm{KG}}(\omega)}\right], \tag{1}$$

where we introduced the dynamic density response function scaled by $v(q) = 4\pi/q^2$. We note that $\chi^*_{\mathrm{KG}}(\omega)$ does not depend on $\vec{q}$ because the electron density response exhibits a quadratic dependence $\propto q^2$ in the small wavenumber limit for a given fixed configuration of ions [6–9].

From Eq. (1) and the fluctuation-dissipation theorem (Eq. (8) in the main paper), we find the scaled DSF in the Kubo-Greenwood approximation:

$$S^*_{\mathrm{KG}}(\omega) \underset{\vec{q}\to 0}{=\!=} v(q) S_{\mathrm{KG}}(\vec{q},\omega) \equiv -\frac{\hbar^2}{n} \frac{1}{1-e^{-\hbar\omega/k_BT}} \,\mathrm{Im}\left[\chi^*_{\mathrm{KG}}(\omega)\right]. \tag{2}$$

Since the ITCF calculation involves only the frequency integration, taking the limit $\vec{q} \to 0$ in the DSF explicitly is not required. Instead, $S^*_{\mathrm{KG}}(\omega)$ can be used directly to perform the η convergence test in the imaginary time domain using

$$F^*_{\mathrm{KG}}(\tau) \underset{\vec{q}\to 0}{=\!=} v(q) F_{\mathrm{KG}}(\tau) \equiv \int_{-\infty}^{\infty} \mathrm{d}\omega\; S^*_{\mathrm{KG}}(\omega)\; e^{-\tau\omega}. \tag{3}$$

After identifying the η-convergence range, filtering of narrow-band fluctuations can be applied to $S^*_{\mathrm{KG}}(\omega)$. Following this, the imaginary part of the scaled density response function $\chi^*_{\mathrm{KG}}(\omega)$ filtered from narrow-band fluctuations can be computed. The real part of the response function $\chi^*_{\mathrm{KG}}(\omega)$ filtered from narrow-band fluctuations is derived using the Kramers-Kronig relations.

Finally, the data with attenuated narrow-band fluctuations for the dynamic dielectric function and conductivity in the Kubo-Greenwood approximation can be obtained using the relations $\varepsilon^{-1}_{\mathrm{KG}}(\omega) = 1 + \chi^*_{\mathrm{KG}}(\omega)$ and $\sigma_{\mathrm{KG}}(\omega) = \frac{i\omega}{4\pi}\left[1 - \varepsilon_{\mathrm{KG}}(\omega)\right]$.

References

[1] Hu SX, Nilson PM, Shaffer NR, Karasiev VV, Golovkin IE, Gu MF, et al. VERITAS: A density-functional theory-based multiband kinetic model for understanding x-ray spectroscopy of dense plasmas. Physics of Plasmas. 2025 07;32(7):073303. https://doi.org/10.1063/5.0273272. https://arxiv.org/abs/https://pubs.aip.org/aip/pop/article-pdf/doi/10.1063/5.0273272/20606838/073303_1_5.0273272.pdf.

[2] Melton CA, Clay I Raymond C, Cochrane KR, Dumi A, Gardiner TA, Lentz MK, et al. Transport coefficients of warm dense matter from Kohn-Sham density functional theory. Physics of Plasmas. 2024 04;31(4):043903. https://doi.org/10.1063/5.0198005. https://arxiv.org/abs/https://pubs.aip.org/aip/pop/article-pdf/doi/10.1063/5.0198005/19888005/043903_1_5.0198005.pdf.

[3] Schörner M, Bethkenhagen M, Döppner T, Kraus D, Fletcher LB, et al. X-ray Thomson scattering spectra from density functional theory molecular dynamics simulations based on a modified Chihara formula. Phys Rev E. 2023 Jun;107:065207. https://doi.org/10.1103/PhysRevE.107.065207.

[4] Calderín L, Karasiev VV, Trickey SB. Kubo–Greenwood electrical conductivity formulation and implementation for projector augmented wave datasets. Computer Physics Communications. 2017;221:118–142. https://doi.org/https://doi.org/10.1016/j.cpc.2017.08.008.

[5] Hadad RE, Roy A, Rabani E, Redmer R, Baer R. Stochastic density functional theory combined with Langevin dynamics for warm dense matter. Phys Rev E. 2024 Jun;109:065304. https://doi.org/10.1103/PhysRevE.109.065304.

[6] Arista NR, Brandt W. Dielectric response of quantum plasmas in thermal equilibrium. Phys Rev A. 1984 Mar;29:1471–1480. https://doi.org/10.1103/PhysRevA.29.1471.

[7] Moldabekov ZA, Bonitz M, Ramazanov TS. Theoretical foundations of quantum hydrodynamics for plasmas. Physics of Plasmas. 2018 03;25(3):031903. https://doi.org/10.1063/1.5003910.

[8] Moldabekov ZA, Shao X, Pavanello M, Vorberger J, Dornheim T. Nonlocal vs local pseudopotentials affect kinetic energy kernels in orbital-free DFT. Electronic Structure. 2025 mar;7(1):015006. https://doi.org/10.1088/2516-1075/adbf5a.

[9] Moldabekov ZA, Shao X, Pavanello M, Vorberger J, Graziani F, Dornheim T. Imposing correct jellium response is key to predict the density response by orbital-free DFT. Phys Rev B. 2023 Dec;108:235168. https://doi.org/10.1103/PhysRevB.108.235168.
